# Community Gardening as a Way to Build Cross-Cultural Community Resilience in Intersectionally Diverse Gardeners: Community-Based Participatory Research and Campus-Community-Partnered Proposal

**DOI:** 10.2196/21218

**Published:** 2020-10-07

**Authors:** Angie Mejia, Manami Bhattacharya, Joshua Miraglia, The Village Community Garden & Learning Center

**Affiliations:** 1 Center for Learning Innovation University of Minnesota Rochester Rochester, MN United States; 2 Division of Health Policy and Management University of Minnesota Minneapolis, MN United States; 3 Research Information Systems Office of the VP for Research University of Minnesota Minneapolis, MN United States; 4 see Acknowledgements

**Keywords:** community gardening, resilience, food insecurity, racial and ethnic minority populations, campus-community partnerships, CBPR

## Abstract

**Background:**

Community-based agriculture has been found to decrease food insecurity and alleviate health inequities. Furthermore, it provides a sense of ownership, resources to help integrate new communities, and a space to nurture existing cultural identities for intersectionally diverse gardeners. This sense of belonging in connection with access to growing plots has been linked to psychological well-being and resilience. However, little is known about how the psychosocial benefits of plot ownership affect resilience and which aspects of this resilience are salient.

**Objective:**

This community-based participatory research (CBPR) project will examine the role of community gardens in decreasing food insecurity and facilitating various forms of resilience in food-insecure groups in Rochester, Minnesota. Since participation in community gardens nurtures various forms of resilience along individual, group, and community dimensions, our research seeks to understand how dimensions of resilience vary along intersectional lines. In addition to mapping the psychosocial benefits linked to plot ownership, we find that examining which forms of resilience are fostered in community-based agricultural projects addresses an important gap in the academic literature. This can help us propose policy-level practices that reduce health inequities connected to food and nutrition at the local level.

**Methods:**

Using a mixed methods approach, this ongoing community-campus partnership will examine the experiences of current and new plot owners. As a CBPR project, our data collection plan, from design to dissemination, incorporates the intellectual and creative labor of the individuals representing members of the campus community (ie, college students and faculty members engaged in other citizen science projects hosted by the garden), community growers, individuals involved in the community garden’s board, and representatives of various organizational bodies. Data collection activities will consist of surveys, in-depth interviews, and photovoice.

**Results:**

This project was funded in January 2020 and approved by the University of Minnesota's Institutional Review Board in March 2020. For the 2020 growing season, we will conduct evaluative interviews about the effect of COVID-19 on community gardeners, including their experiences during this growing season. For the 2021 growing season, data collection, via pre- and postsurveys, is projected to begin in March 2021 and end in November 2021. We will also conduct in-depth interviews from January to April 2021. Data analysis will commence in April 2021. Photovoice activities (ie, data collection, analysis, synthesis, and dissemination) are expected to take place during the spring and summer of 2021.

**Conclusions:**

Findings emerging from this study will provide the preliminary data to foreground community gardening projects and initiatives to improve physical and mental health outcomes in food-insecure communities. Also, the data collected will highlight the role of CBPR methods in disseminating information about the organizational practices of the community garden; this will assist others in planning and implementing similar projects.

**International Registered Report Identifier (IRRID):**

PRR1-10.2196/21218

## Introduction

### Background

Food insecurity is a social condition where at least one member of a household unit has limited access to adequate amounts of nutritious and culturally adequate food or experiences hunger due to lack of economic and other social resources [1]. Food insecurity affected around 14.3 million people in the United States in 2018 [2].  The United States Department of Agriculture (USDA) indicated that half of the populations experiencing this deficit are those categorized as US communities of color [3]. Furthermore, households headed by a foreign-born adult tend to experience more food insecurity than other populations in the United States [4]. Differential access to sources of nutrition “leads to nutritional inequalities and diet-related health inequities in rich and poor cities alike” [5]. Participating in governmental and other community-based food supplementation and nutrition programs, such as food pantries; tapping into informal networks of friends and families for help; developing strategies to access lower-cost food items; regulating eating patterns; and using supplementary sources of income are in the range of strategies employed by food-insecure families in the United States [6].

Community gardening, defined as the use of “plots of land used for growing food by people from different families, typically urban dwellers with limited access to their land” [7], is one such strategy used by families facing food insecurity [8]. Community gardens provide spaces for people with little-to-no access to fresh and nutritious food [9], which may also have an impact on health inequities [10].  Research indicates that community garden access allows people to develop healthy behaviors, practices, and habits that could decrease health inequities in the long run [11], such as increased consumption of fruits and vegetables [12], aiding in obesity prevention [13,14], and engagement in sustained physical activities, such as walking [15].

In addition to a decrease in food insecurity and nutrition-based health inequities, community gardens provide additional related benefits to gardeners, especially those from diverse backgrounds of race, ethnicity, language, class, and other identity markers. Since these spaces are known to operate at different levels of “ownership, access, and degree of democratic control” [16], access to community garden plots nurtures ownership and belonging among refugees, immigrants, and other marginalized and minoritized groups in areas where they are socially devalued [17,18]. As in the case of US foreign-born groups, community gardens are also uniquely situated to both facilitate integration within new communities [18-20] and provide a context where these groups can continue to nurture existing cultural identities and practices [21,22]. This sense of belonging in connection with access to plots [18] has been linked to psychological well-being and resilience [7]. In addition to sustaining spaces for the exchange of knowledge and the creation of community bonds, these spaces might serve as “physical havens for safety and the development of social and spiritual support” [23] in areas where racial or ethnic and other minorities tend not to be welcomed by the majority population.

While there have been a few studies looking at the role of community gardens in fostering food security, well-being, and resilience in marginalized groups [7,24,25], especially during times of economic uncertainty [26], several questions remain unanswered. Despite gardens usually being located in neighborhoods populated by racial and ethnic minorities, community gardens tend to be most accessed by Anglo White gardeners [27]. Furthermore, community gardens have been conceptualized as “socio-ecological refuges” [23,28,29], since they increase social cohesion [30] and feelings of empowerment [31,32] in historically marginalized and socially devalued people by belonging to groups deemed mainstream in US society. However, there is little knowledge of how the various psychosocial benefits [33,34] of plot ownership that affect resilience in marginalized communities and which aspects of resilience are salient and possible by this process.

### Objectives and Research Aims

#### Overview

The overall objective of this project is to understand the role of community gardens in decreasing food insecurity and facilitating psychosocial wellness and resilience among minoritized communities in Southeast Minnesota. The motivation for this inquiry is that the psychosocial benefits of community gardening include nurturing various forms of resilience in US gardeners belonging to racial and ethnic, refugee, and immigrant communities. In particular, our research will attempt to answer three interconnected issues: (1) the role of community gardens in *decreasing food insecurity* among community gardeners of refugee, immigrant, or racial and ethnic minority backgrounds, (2) the role of community gardening in *self-reported emotional health and well-being* in community gardeners from refugee, immigrant, or racial and ethnic minority backgrounds, and (3) the role of community gardening in *increasing resilience* at three levels—individual, group, and community dimensions—in community gardeners of refugee, immigrant, or racial and ethnic minority backgrounds. The rationale of this proposed research is that, in addition to mapping the psychosocial benefits linked to community garden plot ownership, examining which forms of resilience are connected to community gardening addresses a gap in the academic literature, which can help us propose policy-level practices that reduce health inequities connected to food and nutrition at the local level.

Adhering to a community-based participatory research (CBPR) process, the specific aims outlined below will help us gather data for a larger examination of the role of community gardening in shaping health across minoritized communities in Minnesota. We will map and analyze these unique patterns through interviews and pre- and postsurveys of food-insecure populations in Rochester, Minnesota: current and new growers assigned community garden plots.

#### Aim 1

Our first aim is to examine food security in different, underserved communities in Rochester, Minnesota. Racial or ethnic, sociocultural, and demographic disparities in food security do not always align with data at the aggregate level or are masked when examined. Our pre- and postsurveys include measures on food insecurity, as detailed in our Methods section, and our qualitative interviews will also explore gardeners’ experiences and perceptions of food insecurity and access in their community.

#### Aim 2

Our second aim is to identify the psychosocial benefits linked to community garden plot ownership. Access to community plots has been found to have psychological benefits for gardeners. Also, these are spaces that facilitate cross-cultural knowledge sharing [34-36]. We will examine these psychosocial benefits in both our pre- and postsurveys and qualitative interviews, as well as in our photovoice interviews with plot owners.

#### Aim 3

Our third aim is to establish the extent to which community garden plot ownership and support by *The Village Community Garden & Learning Center* (VCGLC) staff and volunteers facilitate resilience in food-insecure members of US racial or ethnic, immigrant, or refugee groups in Rochester, Minnesota. We will measure resilience by conducting interviews and pre- and postsurveys with plot owners. Our survey includes replication of an instrument developed by Kimhi and colleagues [37] to examine three different dimensions of resilience in majority-minority groups—individual, community, and ethnic origin–based resilience—taking into consideration protective and suppressing factors that shape a sense of resilience.

## Methods

### Target Community

Current research indicates that communities made up of refugees [4], immigrants [38], and racial or ethnic minorities [39] are at a higher risk of being food insecure than other population groups. Pilot data from conversations with VCGLC coordinators and board members collected at the end of the 2019 growing season suggest that a great need for the growers is being met. Most of these individuals are of Southeast Asian descent (ie, Hmong, Cambodian, and Indian), are Latino and Latina, are of African diaspora ancestry, or are individuals coming from economically fragile or working-class backgrounds. Racial and ethnic minorities and those claiming refugee status make up more than 90% of the current growers. At the moment, the health department is currently working with others in the community, including members of the VCGLC board, to conduct other assessment-related studies looking at food security in Rochester, Minnesota, to get a better understanding of the issue, specifically related to communities of color.

### The Village Community Garden & Learning Center

As a community-campus initiative, the VCGLC maintains community garden spaces for diverse Rochester groups to feed themselves, reduce food costs, and foster connections across cultures and experiences. Since its inception, the VCGLC has served as a community partner and learning site for an upper-division course at the University of Minnesota Rochester. In a year and a half, the VCGLC has increased access to fresh, healthy, and culturally relevant foods for racial or ethnic minority, immigrant, and refugee community members who have limited access to such foods. In addition to supporting over 120 intersectionally diverse growers, the VCGLC currently donates excess vegetables and fruits to local food pantries. Stakeholders with a firm commitment to the VCGLC project, via funding and other forms of support, include higher education, the local public health office, the water and soil conservation district office, the local library, the mayor’s office, various food cooperatives, nonprofit organizations, and the local farmers’ market.

### Community-Based Participatory Research

Our partnership’s long-term goal is to improve the health of food-insecure groups in Rochester, Minnesota, via access to community garden spaces that nurture connections and community building among different groups by using equity-based approaches such as CBPR. By CBPR, we refer to the iterative and holistic process outlined by Israel and colleagues [40,41] that sees communities involved as unique units of identity, and centers the particular strengths and resources that these communities bring when defining, implementing, and engaging in research. Collaboration between community members and researchers is embedded throughout the research process, with the knowledge and practices gleaned being used to benefit all involved. As a process sensitive to the ebbs and flows of relationship building and trust, it also aims to disseminate knowledge and practices with language that is empowering and accessible to the communities involved, while being aware of the power dynamics inherent in research.

This project utilizes a community-participatory-based research oversight board—referred to throughout this article as the VCGLC board—composed of community members who are current growers in the garden, students, faculty researchers, and representatives from key local entities. Furthermore, many CBPR teams tend not to reflect the diversity of the community at the center of their inquiry; however, our board makeup not only reflects the membership of the garden but also features the collaboration of two research project supervisors with experience in academic-community research partnerships and community health work with ethnic and racial minorities. Furthermore, both the community principal investigator and the academic principal investigator are first-generation Americans and native-language speakers of the two represented refugee and immigrant communities in the garden. Also, the research data will be collected by research assistants that speak the language, some of whom are members of the communities represented in the garden: Cambodian, Latino and Latina, Hmong, and Somalian. We will also train and compensate students and members of our board, many of whom are also community gardeners, in collecting, transcribing, and analyzing in-depth interviews and conducting some of the pre- and postsurveys.

### Data Collection

COVID-19 has created and increased the severity of social issues that cause food insecurity, economic insecurity, and disconnection. Therefore, we believe that evaluating the outcomes of community-engaged gardening would not provide a usual, valid result. We have chosen to outline an evaluation plan for this COVID-19 growing season (2020) and a research plan for the next season (2021).

### COVID-19 Growing Season (2020)

In the fall 2020 growing season, we plan to conduct a qualitative evaluation program and gather responses regarding COVID-19-related difficulties. Open-air interviews will be conducted in the community garden. Participants and moderators will be masked and will maintain adequate distancing. From September to November 2020, we will conduct 20 interviews, each 60 minutes in length, with the majority of the gardeners in English, Spanish, and Khmer. Participants will be recruited via telephone, an established means of communication for this project. We will sample participants to roughly match interviewees with the composition of our gardeners: approximately 10 Cambodian people, 5 Hispanic people, and 5 participants randomly sampled from other groups, the majority of whom are also of minority backgrounds. Each participant will be offered a US $20 gift card to a local grocery store for completing the interview.

These semistructured qualitative sessions will focus on evaluating the logistics of participating in the community garden, such as sign-up, plot allocation, provision of supplies, and support by the VCGLC staff throughout the growing season, as well as evaluating the community reaction (eg, we will ask if the community garden meets the participants’ needs) and eliciting ideas to strengthen and expand the program. Finally, we will ask questions about how the community garden was accessed in relation to COVID-19. Some queries will involve perceptions of how the community garden helped participants navigate COVID-19-related economic difficulties, including food security, as well as other difficulties like the ability to grow food that may have been unavailable due to grocery store closures. We will also ask participants if the garden helped with other difficulties connected to the pandemic, such as social isolation and stress from stay-at-home orders.

At the time of writing, we are meeting as a board to develop the in-depth interview questions in light of COVID-19. Collected data will be transcribed; transcripts will then be coded inductively and deductively by at least two coders, using NVivo software (QSR International). Interrater reliability between coders will be checked.

### Next Growing Season (2021)

We will measure food insecurity, emotional health, and resilience by employing a mixed methods approach, including pre- and postsurveys and semistructured interviews with plot owners.

#### Survey Data Collection

During the first months of the project, we engaged the VCGLC board to give us direction in refining data collection tools to measure the above outcomes. As of June 2020, the board discussed and approved the following measures to be used to survey gardeners at the start (ie, June 2021) and at the end of the growing season (ie, October 2021). We will conduct up to 130 preseason surveys and 130 postseason surveys of the same gardeners. Current enrollment is 130 plots, meaning that we plan to survey everyone involved. This number may increase next year, in which case we will use stratified random sampling so that the final sample reflects the ethnic makeup of the gardeners, which we learned from our community garden contract that we administered preseason. Participants will be recruited by telephone. Surveys will be administered via phone; non-English speakers will be given the survey in their native language. We anticipate high response rates due to the community’s engagement with programmatic and community activities. Each participant will be compensated with a US $10 gift card for completing the pre- and postsurveys.

#### Measures

Food insecurity will be measured using the USDA’s US Household Food Security Survey Module and the Food Insecurity Experience Scale Survey Module [42]. This scale not only measures severity levels of food insecurity arising from lack of resources, but it has also been cross-culturally validated using the language represented by the VCGLC’s growers*.* We will include items regarding food behaviors from the US Centers for Disease Control and Prevention’s Behavioral Risk Factor Surveillance System [43]. Psychosocial and resilience measures include the Kessler Psychological Distress Scale [44], the Connor-Davidson Resilience Scale [45], the Conjoint Community Resiliency Assessment Measure, and the short National Resilience Scale [46]. Many of these measures include validated translations in the languages of the growers and members of the VCGLC board. For those that do not, we will translate and back-translate de novo. So far, we have translated the instruments into Spanish and Khmer; we have plans for other languages as needed.

#### Qualitative Data Collection

##### Interviews

By conducting in-depth interviews with plot owners and people who attend our events, we will investigate the role of the VCGLC in facilitating opportunities and providing resources for cross-community connections, furthering a sense of collective well-being and increasing the sense of belonging and empowerment. Conditions willing, we will conduct in-depth interviews from October to December 2021 with current and incoming gardeners. Interviews will be conducted in the participant’s preferred language with one of the members of the VCGLC board interpreting or facilitating, some of whom are native speakers of Khmer, Spanish, and Somali. A telephone interpreter will be hired from one of our local community partners for other languages. Participants will be recruited via phone or on-site at the garden. Interviews will be 60 minutes long, semistructured, and conducted on-site or via phone. Each participant will be offered a US $20 gift card to a local grocery store for participating in the interview.

##### Photovoice

For the next growing season (2021), we will train a select group of the VCGLC’s gardeners in photovoice, a visual participatory action research approach that gives voice to marginalized communities by using pictures to present their concerns to stakeholders [47,48]. Participants and facilitators will each be paid a stipend for participating in the photovoice meetings. This part of the research project will add a visual component that can be used during the community dissemination event at the end of 2021. An aspect of photovoice is the hosting of a photo exhibit to present findings to community stakeholders. This event will be open to growers and their families as well as to the wider community for input and support on the VCGLC’s future steps.

##### Campus-Community Partnership

Community collaborations between educational institutions and community-based agricultural bodies strengthen community-campus relationships [49]. Using a model like CBPR, which puts equal weight on community and academic members’ research interests, knowledge production, and opinions about how the research should be conducted, can promote healing and trust between the community and the University of Minnesota Rochester.

### Data Analysis

We plan to use chi-square tests and paired *t* tests of the scales’ scores to compare pre- and postresponses. This will give us an understanding of whether participants indicated that community gardening would improve food security, emotional health, and resilience. Qualitative interviews will be inductively and deductively coded by at least two coders; an interrater reliability score will be calculated.

A note on mixed methods: we propose an approach where data from the qualitative methods, including our photovoice findings, will be used to understand the experience of food insecurity, resilience, and psychosocial well-being as related to community gardening participation. The collection of quantitative data and in-depth interviews is happening almost simultaneously to allow both the previously collected 2020 evaluation data and the analytical insights from our 2021 surveys to inform the qualitative analysis of in-depth interviews and the photovoice process. Quantitative pre- and postsurvey data will allow us to track changes in our outcomes using validated scales across nearly all participants. Our qualitative data will allow us to better understand how and why gardeners felt that the process of gardening and community engagement affected them. While our quantitative data will allow us to compare outcomes effectively, the qualitative data will allow us to retain the ideas and voices of gardeners spoken in their own words.

## Results

### Overview

This project was funded in January 2020 and approved by the University of Minnesota's Institutional Review Board in March 2020. As of September 2, 2020, we have begun recruiting participants for the evaluation interviews. In addition, our original research design had to change due to pandemic conditions.

### COVID-19 Safety Protocols

Our board met and approved a safety protocol and the creation of culturally relevant signage. These promote and inform how to socially distance while using the garden. We have also developed an approved calendar system to decrease contact between gardeners.

### Increase in Participants

Initially, we sought to enroll all 30 of the active community gardeners. As of the time of writing, our total number of participants increased from 30 to 130, as noted in the Methods section, not including gardeners wait-listed to receive a plot. This was the result of two interconnected issues affecting the community at large: first, the closing of other community garden spaces once supported by a local social service agency; and second, the increase of people reaching out to the community garden’s founder—a long-standing leader and member in the Southeast Asian community—for help finding places to grow food, citing fears that the pandemic would cause ethnic-based markets of culturally specific produce to close. The board sought areas for this sudden influx of participants by meeting with faith-based organizations, social services, and other agencies, one being the local museum, that could provide adequate space for garden plots. At the time of updating this proposal we have four sites to manage. We will survey all of the people currently assigned a garden plot in these four sites, but the board is currently meeting to decide how we will sample for the qualitative interviews.

### Research Design

The board indicated the need to capture people’s growing and food needs during a moment of crisis. We are currently meeting to discuss the way we will integrate COVID-19-specific questions during the in-depth interview phase of our research. We have also added to the pre- and postsurveys questions that ask participants how the current health pandemic has affected how they shop for produce.

## Discussion

### Overview

The three research aims detailed earlier in this proposal build toward a community-led design and implementation of a more extensive intervention to promote community-based agricultural initiatives to improve physical and mental health in food-insecure communities of color in Rochester, Minnesota. Accomplishing these aims will shape and refine ongoing practices by the VCGLC that will assist others in planning and implementing similar community garden projects.

### Limitations

There may be some limitations to our study, especially when taking into account the CBPR approach to the project, recruitment, and COVID-19. Firstly, our research plans may shift, not only because CBPR-based initiatives take more time, but because of input from community stakeholders. One example would be that additional time spent by research assistants requires additional compensation. Another shift may happen when members of the board seek to implement changes from one methodological approach, say photovoice, in response to already-collected data.

Due to our strategy of going through community leaders who have experience working in community gardens, owning and managing small farms, or designing urban green spaces, some of the people recruited might have more experience. Thus, results from this project may not be generalizable to all types of community garden projects, especially the few that serve growers from marginalized backgrounds.

Also, just like past research [6,50] and conversations with the VCGLC board suggest, COVID-19 worries may force people to strategize ways to minimize their food insecurity (eg, eating less, borrowing more money to buy food, and stretching food supplies to last longer). Since some of our participants were recruited with the help of a food pantry, some of the people agreeing to sign up might have experienced food insecurity in the past. Newly food-insecure families may deal with pandemic effects differently than those who have experienced bouts of food insecurity before COVID-19 [51], which could mean that we might not be capturing the experience of newly food-insecure community garden growers.

### Strengths

Despite the limitations listed above, our project has several strengths: firstly, the makeup of the VCGLC board (ie, over 50% of the members represent not only growers but belong to communities found in the garden). Also, the processes outlined in our memorandum of understanding prevent us from replicating issues associated with some CBPR projects, such as “unintended consequences of re-colonizing the population” involved in the research [52]. Furthermore, both the academic investigator and the community coinvestigator identify as second-generation and first-generation immigrants to the United States, respectively, and belong to two of the largest ethnic and racial communities represented by the growers. Due to the time we spent carefully developing our CBPR processes and the sensitivity to the dynamics of power and privilege, our project might avoid issues that make it difficult for other CBPR projects to fulfill their goals of an equal research partnership that could “[e]nable fairness and equality at each step of the research” [53].

COVID-19 is intensifying inequities, especially concerning food and nutrition. For example, these social shifts and responses to the pandemic food issues increase chronic health conditions like those related to obesity [54], which makes our study timely. In the case of obesity, there is not only the link between obesity and food access; this is one of those chronic health conditions that increase the severity of COVID-19 symptoms [55].

Our study is focused on emotional health, which is also relevant to COVID-19 effects. As research suggests, there is a strong association between depression and anxiety and pandemic-related cases of food insecurity [56]. Also, there is what we call the urgency and agency of place during crisis, meaning that the garden may be giving growers a sense of empowerment at a moment of uncertainty, with the added caring support of our community garden volunteers and board members. Research has shown that gardens are considered safe spaces that facilitate positive mental health for those whose cultures are devalued by mainstream society. Our garden is providing this and more to growers from minoritized communities when they might bear the brunt of indignities and negative social effects.

### Conclusions

Findings emerging from this study will provide us with preliminary data to implement a more extensive intervention for community gardening projects and initiatives to improve physical and mental health outcomes in food-insecure communities. Also, data collected will help us highlight the role of CBPR methods in disseminating information on the organizational community garden practices that can assist others in planning and implementing similar projects.

## Abbreviations

CBPR: community-based participatory research
USDA: United States Department of Agriculture
VCGLC: The Village Community Garden & Learning Center
